# A Model for an Online Learning Management System for Simulation-Based Acquisition of Psychomotor Skills in Health Professions Education

**DOI:** 10.7759/cureus.14055

**Published:** 2021-03-23

**Authors:** Adam Dubrowski, Bill Kapralos, Eva Peisachovich, Celina Da Silva, Andrei Torres

**Affiliations:** 1 maxSIMhealth, Faculty of Health Sciences, Ontario Tech University, Oshawa, CAN; 2 Software Informatics Research Centre, Ontario Tech University, Oshawa, CAN; 3 Medical Education and Simulation, York University, Toronto, CAN; 4 Computer Science, Ontario Tech University, Oshawa, CAN

**Keywords:** simulation, on-line, practice, learning, virtual, video

## Abstract

The current coronavirus disease (COVID-19) pandemic has shifted traditional educational approaches in health professions education (HPE) from in-person to remote learning. Although pedagogical strategies have been developed and implemented rapidly to support cognitive and affective domains of learning in HPE, less progress has occurred in psychomotor skills acquisition. Psychomotor skills, referred to as technical skills training, are underpinned by educational theories and conceptual frameworks. Considering the widening gap in learning domains, this editorial provides an overview and recommendations for developing and implementing remote training supported by educational theories, such as deliberate practice, and conceptual frameworks in technical skills acquisition in HPE. We begin by discussing the unique curricular needs for remote psychomotor skills in medical teaching-learning contexts and subsequently present a theory-driven and evidence-based model for remote psychomotor skills acquisition.

## Editorial

Introduction 

Since coronavirus disease (COVID-19) was declared a pandemic by the World Health Organization on March 11, 2020, educational institutions worldwide have witnessed an abrupt move from traditional face-to-face to remote learning. Higher education institutions dedicated to health professions education (HPE) have experienced a similar and rapid shift from in-person learning to remote synchronous and asynchronous learning.

The case of psychomotor skills learning in HPE

A well-developed HPE program, with in-person or remote pedagogical approach, must address three domains of learning: cognitive (thinking), affective (emotions or feeling), and psychomotor (physical or kinesthetic) [1]. The cognitive domain aims to develop skills and knowledge acquisition and encompasses six categories: knowledge, comprehension, application, analysis, synthesis, and evaluation. The affective domain includes the feelings, emotions, and attitudes of the learner. Whereas, the psychomotor domain addresses motor and coordination skills involving perception, mechanism, complex overt response, adaptation, and origination. 

Given the unique educational approaches to each of the domains of learning, creating a single remote, online learning management system (LMS) to support all would be impossible. For the purpose of this discussion, an LMS is a software application for the administration, documentation, tracking, reporting, automation, and delivery of educational courses, training programs, or learning and development programs. Consequently, our thesis, as articulated in this editorial, is that there is an immediate need for a unique, remote, online psychomotor skills acquisition LMS. 

For decades, the overarching theory that has been used to guide the development of psychomotor skills in HPE programs is deliberate practice [2]. Deliberate practice refers to a particular type of practice that is purposeful and systematic. While regular practice may include repetitions of actions, deliberate practice requires repetition, motivation, consistency, accurate feedback, all aligned with a single goal of improving future performance [2]. 

**Figure 1:**
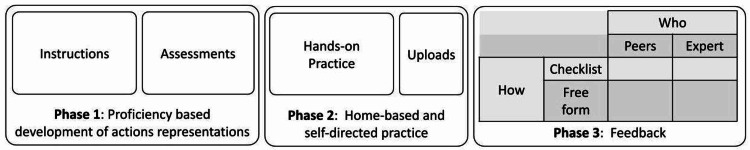
Model illustrating three phases of learning and the components required for the psychomotor skills learning management system In Phase 1, the learners need to be provided with active instructions that should include a set of assessments. For example, passive instructions are videos of performance, such as those found on YouTube. In contrast, active instructions would be a set of videos that highlight error-free, erroneous, as well as alternative ways of performing the skills. The learners need to review these videos and use assessment rubrics to spot the errors and alternatives.  To enable proficiency-based training, the educator or course manager could set proficiency criteria that allow the learner to progress to the next phase only if they spot a certain, predetermined amount of errors or alternatives. Once proficient action representations have been formed, the learners move to Phase 2. Here, they would practice at home using simulators specifically designed for this purpose. They can go back to the instructional materials for more guidance, and once satisfied with their performance, they video-record a test trial and upload it to the LMS for feedback. In Phase 3, the learners will receive feedback on their test trial from either their peers or an expert or a combination of both. The feedback can be based on checklists, such as Objective Structured Assessment of Technical Skills, or the feedback can be delivered in free form by providing comments. Utilizing peers for assessment purposes expands the learning opportunities as the learners who provide feedback will engage in observational practice and error detection.

We propose a theoretical, evidence-based model to structure remote, online psychomotor skills acquisition LMS illustrated in Figure 1, which illustrates four main components of such LMS, associated with the four elements of deliberate practice, specifically a) opportunities for hands-on repetition; mechanisms to increase b) motivation and c) consistency in the use of the LMS; and d) provision of accurate feedback. One key component that is necessary within this context is the ability to engage in hands-on practice, which is one of the most significant differences between remote, online LMSs designed for the acquisition of psychomotor skills and those developed to acquire cognitive and affective skills. There are several ways in which this can be accomplished, from take-home task trainer simulators to purchasing off-the-shelf simulators to "do it yourself" solutions. 

However, practice at home may result in unintended learning outcomes, such as the development of bad habits. Therefore, instructions evoking active learning strategies, increasing motivation to practice, and feedback are essential to be embedded within the remote, online psychomotor skills acquisition LMS. 

Motivation

Developing proficiency in any skill may be difficult and monotonous. Motivation is a crucial element to overcome the frustration with failures and periods of slow progress, especially when learners are practising from home or a remote location without direct supervision or peer learners. One way to increase motivation is by setting specific, realistic goals [3]. These goals need to consider the learner's current knowledge and skill level and push those boundaries little by little, consistently expanding their capacity to perform [3]. 

In addition to setting goals, the learner must push past "trying harder" to "trying differently." Thus, the goals, often encapsulated as learning objectives and instructions, should motivate learners by providing variability in approaches, such as demonstrating variations in techniques and technologies that are typically used in practice (also known as the “tricks” and “shortcuts”). 

Another good example is to demonstrate the correct and the less correct way of performing the skill and tasking the learner to pick the sub-optimal demonstrations through quizzes. One powerful technique described by Cheung et al. [4] is to use a combination of computer-based video instructions (CBVI) and "spot the difference" quizzes [4]. Specifically, when coupled with forced identification of differences through checklist-based quizzes, CBVIs showing variable performances engage in deeper levels of analysis, hypothetically resulting in more profound levels of learning. 

Consistency

One of the foundational aspects of deliberate practice is consistency. However, it has been documented that remote learners often fail to engage in such a practice regimen [5]. For example, Cheung et al. [4] employed an "Observational Practice and Educational Networking" (OPEN) LMS to develop skills related to central venous catheterization (CVC), a complex clinical skill for novice medical learners. What the researchers found was that although using the OPEN resulted in an increased capacity to perform the CVC skill, the analysis of the online learning activities revealed that despite the learners being instructed to consistently interact with the OPEN system for one week before testing, they were predominantly active on the first and the last day of the practice period. This was taken as evidence that more significant incentives were required to address the need for training consistency. 

Gamification may provide a solution. For example, the implementation of "Reddit-like” elements, whereby peers rate the quality of others comments or interactions may improve consistency. Leaderboards, a form of social comparative feedback component that provides learners with information regarding how well they are doing concerning their peers can also be used. Such comparative information may be provided both individually and in a general context by showing the learners position (i.e., ranking scores) on a private individual leaderboard (e.g., "Forum likes: #2"), ensuring that the learners do not have access to the scores of their peers, thus avoiding comparisons that could be detrimental to motivation. Finally, module division in the form of a segmented progress bar may allow learners to track their progress in each course and each particular course component and can improve consistency as well as motivation. 

Feedback

In addition to the amount of practice, feedback is essential for identifying areas for improvement and obtaining a realistic view of progress [2, 3]. Whether one-on-one coaching with a teacher, mentor, or peer or some form of self-assessment, the learner requires a means of pinpointing strengths and weaknesses. A two-by-two matrix of feedback is illustrated in Figure 1 Phase 3. 

Specifically, the remote, online psychomotor skills acquisition LMS can incorporate a combination of peer-to-peer and expert-based feedback, guided by checklists or free form text. There are advantages and disadvantages to these approaches, although there is no clear evidence of which method - peer-to-peer or expert - is more effective [4]. 

**Figure 2:**
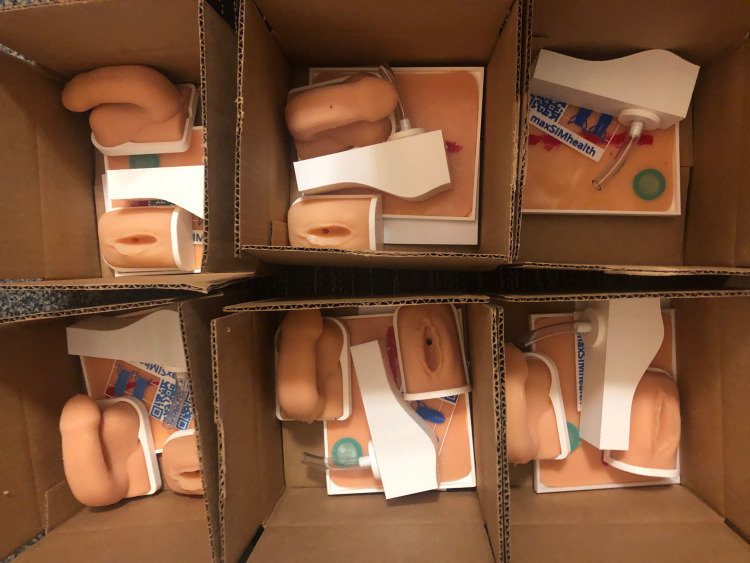
Packages of simulators that were mailed out to each of our 175 nursing students for home-based practice

One essential feature that must be embedded within a remote, psychomotor LMS is uploading practice attempts to the system for feedback. Based on what we presented thus far, we propose that the learner video records the "proficient" attempt and uploads it to the LMS to receive constructive feedback. In one of the pioneering studies, Jowett et al. [5] asked the question whether junior medical trainees can learn suturing skills to the same degree when using computer-based video instructions and self-directed learning as they do when instructed by experts during a face-to-face, time-based learning session in a simulation laboratory. The results of the Jowett et al. study revealed that students who engaged in self-directed learning (i.e., they could stop practice whenever they "felt" like they were proficient) were as skilled as students who were forced to practice more traditionally. Furthermore, moving the self-directed learners to practice their skills more (extra practice after they felt they were proficient) did not result in additional learning. These findings suggest that it is feasible to allow learners to practice some skills at home, and only when they feel that they are competent they can upload their performances for subsequent feedback from both experts and peers. One approach that can be used to achieve this self-assessed performance would be through the use of checklists and global rating scores [5]. Anecdotally, during the COVID-19 pandemic, when our simulation lab closed, we have supplied each of our 175 first-year nursing students with take-home simulators for male and female catheter insertions (Figure 2), wound cleaning, and intravascular access. This resulted in the production of nearly 700 simulators at the cost of $6,000 (Canadian) for the program. Subsequent course evaluations showed high levels of satisfaction with the simulators; however, the students actively sought an LMS that would support their practice.

Summary

Contrary to common belief, it is not just practice that makes perfect, but rather a well-designed and deliberate practice model that makes perfect [3]. Although for many decades we have witnessed a gradual shift to remote, online delivery of many educational programs, including HPE, the recent pandemic catalyzed this transition from face-to-face to online training. 

In this editorial, we argue that although there may be a large overlap in how we design remote, online learning environments for the development of cognitive, affective, and psychomotor skills, there are distinctive theories, frameworks, and models that support psychomotor learning that must be incorporated to create practical and effective remote learning experiences. 

Consequently, here, we have outlined one of the most prevailing theories - deliberate practice - and operationalized how remote psychomotor skills acquisition LMS could be designed to make these most effective.
